# Transthyretin Misfolding, A Fatal Structural Pathogenesis Mechanism

**DOI:** 10.3390/ijms22094429

**Published:** 2021-04-23

**Authors:** Jin-Beom Si, Bokyung Kim, Jin Hae Kim

**Affiliations:** Department of New Biology, Daegu Gyeongbuk Institute of Science & Technology (DGIST), Daegu 42988, Korea; susilia@dgist.ac.kr (J.-B.S.); bkkim0925@gmail.com (B.K.)

**Keywords:** transthyretin misfolding, transthyretin amyloidosis, protein misfolding, amyloid

## Abstract

Transthyretin (TTR) is an essential transporter of a thyroid hormone and a holo-retinol binding protein, found abundantly in human plasma and cerebrospinal fluid. In addition, this protein is infamous for its amyloidogenic propensity, causing various amyloidoses in humans, such as senile systemic amyloidosis, familial amyloid polyneuropathy, and familial amyloid cardiomyopathy. It has been known for over two decades that decreased stability of the native tetrameric conformation of TTR is the main cause of these diseases. Yet, mechanistic details on the amyloidogenic transformation of TTR were not clear until recent multidisciplinary investigations on various structural states of TTR. In this review, we discuss recent advancements in the structural understanding of TTR misfolding and amyloidosis processes. Special emphasis has been laid on the observations of novel structural features in various amyloidogenic species of TTR. In addition, proteolysis-induced fragmentation of TTR, a recently proposed mechanism facilitating TTR amyloidosis, has been discussed in light of its structural consequences and relevance to acknowledge the amyloidogenicity of TTR.

## 1. Introduction

Transthyretin (TTR) is a transporter of thyroxine (T_4_, a thyroid hormone) and a holo-retinol binding protein, from which its name has originated (TRANSporter of THYroxine and RETINol) [1,2,3]. TTR is abundant in the human plasma and cerebrospinal fluid (CSF). In plasma, it is mainly produced in the liver at 3–5 μM, whereas in CSF, it is mostly made in the choroid plexus at 0.25–0.5 μM [4,5]. TTR is the first and the second major transporter of T_4_ in human CSF and plasma, respectively [6]. TTR production in the retina and pancreas has also been reported [7,8].

**Figure 1:**
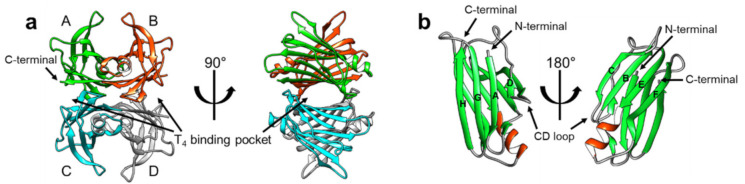
The X-ray crystallographic structural model of transthyretin (TTR) in its native-like (tetrameric) state (PDB 2QGB). (**a**) The tetrameric structure of TTR has been shown in two orientations, with the T_4_-binding pockets marked with arrows. The monomeric subunits are denoted by ‘A’ through ‘D’ and colored differently for better visualization. Note that the TTR tetramer has two dimeric interfaces; the interface between AC and BD is maintained by an extensive network of hydrogen bonds, whereas the interface between AB and CD is mainly constituted by hydrophobic interactions. (**b**) The monomeric subunit of the tetrameric complex (**a**) is displayed from two different orientations to show its secondary and tertiary configurations. Note that the eight β-strands are marked by ‘A’ through ‘H’ according to their sequential order from the N-terminal, showing the DAGH and CBEF β-sheets.

TTR has a β-strand-rich secondary structure; eight β-strands form two β-sheets (CBEF and DAGH), stacking with each other to establish a β-sandwich tertiary structure, with a short α-helix between the β-strands E and F (Figure 1). Its functionality as a T_4_ transporter originates from the quaternary structure. In its native state, TTR forms a tetrameric complex, giving rise to two hydrophobic pockets for binding T_4_ (Figure 1) [9,10]. The tetrameric architecture of TTR is maintained as a dimer of dimers; one dimeric interface between the subunits AC and BD is maintained by an extensive hydrogen-bond network involving the β-strands F and H, whereas the other dimeric interface between the subunits AB and CD is stabilized mainly by hydrophobic interactions (Figure 1a), thus, efficiently constituting the optimal binding pockets for T_4_ [11]. Notably, most structural models of TTR (Figure 1) were determined by X-ray crystallography, indicating that structural heterogeneity, which may exist in a physiological condition, could not be properly reflected to these models due to the crystal symmetry and packing artifacts.

In addition to its physiological functions, TTR has also attracted attention owing to its pathological features of aggregating and forming amyloid fibrils. Since the identification of TTR in human amyloid deposits in the 1970s [12], TTR amyloidosis has been shown to be strongly correlated with several amyloidoses. For example, senile systemic amyloidosis is known to be caused by spontaneous aggregation of TTR [13], whereas familial amyloid polyneuropathy (FAP) [14] and familial amyloid cardiomyopathy (FAC) [15] are caused by genetic mutations in TTR. More than 100 TTR mutations have been reported to date, many of which facilitate pathogenic aggregation and amyloid formation [16]. Notably, a recent study estimated that 3% of the African American population has the V122I mutation, which can cause TTR cardiac amyloidosis [17,18], and 8% of the patients suspected to have cardiac amyloidosis were reported to have pathogenic TTR mutations [19].

Many biochemical and biophysical studies have been conducted to investigate the amyloidogenic (amyloid-forming) properties of TTR, and it is generally accepted that the tertiary and quaternary stability of TTR correlates with its amyloidogenic propensity [20]. In particular, in vitro turbidity and thioflavin T fluorescence experiments showed that the tetrameric stability of TTR diminished dramatically under mild acidic conditions, which resulted in its dissociation to monomeric species and the subsequent accumulation of amyloid fibrils [21,22]. It was also reported that a molecule that binds to the T_4_-binding site could stabilize the tetrameric state and suppress the aggregation of TTR efficiently [23]. By conducting extensive biochemical and biophysical studies on the TTR-ligand complexes, Kelly et al. succeeded in developing a ‘kinetic stabilizer’ to stabilize the tetrameric state of TTR and suppress/prevent its aggregation [24,25]. Tafamidis, a kinetic stabilizer designed to target the T_4_-binding site, is an effective drug that is currently approved for clinical use [26,27,28].

However, despite the central role of structural investigations in the development of novel therapeutics, initial structural studies concentrated more on the tetrameric conformation, and provided limited information to reveal the mechanistic details pertaining to TTR aggregation [29,30]. Rather, recent investigations on the structural states of TTR revealed that the protein, either in its stable tetrameric state or in the less stable monomeric state, adopts significant structural heterogeneity, which is strongly associated with many of its physiological and pathological features [31]. Thus, in this review, we recapitulate the recent advancements pertaining to the elucidation of the structural heterogeneity of various oligomeric states in the TTR aggregation pathway. In particular, we discuss the novel proteolysis-induced aggregation mechanism of TTR and the resultant structural deformation that is directly related to TTR pathology.

## 2. Deformation of the TTR Quaternary Structure

**Figure 2:**
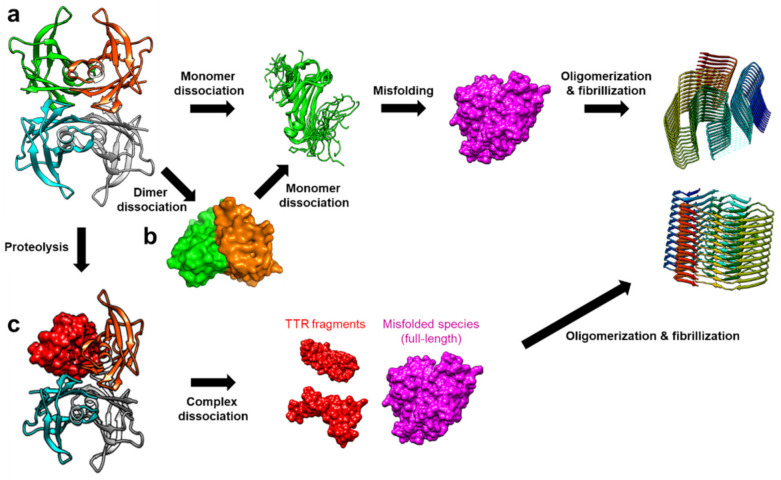
The amyloidosis pathways of TTR. (**a**) Tetramer dissociation into monomers is one of the dominant causes of TTR amyloidosis. The dissociated monomers are more susceptible to misfolding, resulting in aggregation into non-native oligomers and amyloid fibrils [20]. (**b**) Alternatively, it was suggested that TTR dimers are formed as an intermediate state in the tetramer dissociation mechanism [32]. (**c**) Another notable aggregation mechanism was recently proposed to involve proteolysis of TTR, which compromises the tertiary and quaternary stability of TTR, and facilitates the formation of the misfolded monomers. These TTR fragments are more amyloidogenic, thus, aggregating with full-length proteins to form amyloid fibrils even at a physiological pH [33].

TTR amyloidosis is initiated by the dissociation of the tetramer into monomers, which is followed by deformation into misfolded monomers, non-native oligomers, and finally amyloid fibrils (Figure 2) [20]. In this section, recent advancements in revealing details regarding various non-native conformational states of TTR are discussed. Particularly, in addition to numerous structural studies on TTR tetramers, novel findings on the other quaternary states of TTR are discussed focusing on their physiological and pathological aspects.

### 2.1. Conformational States of TTR Monomers

**Figure 3:**
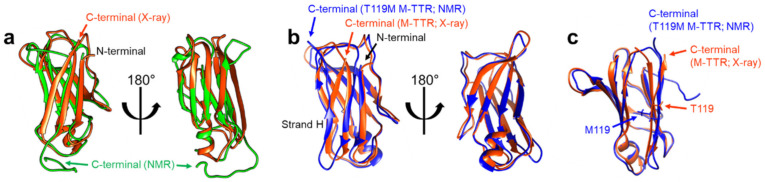
NMR solution structural models of M-TTR (PDB 2NBO [35]) and T119M M-TTR (PDB 2NBP [34]). (**a**) The NMR structural model of M-TTR (the lowest energy conformer; green) is shown with the X-ray crystallographic model of M-TTR in the tetrameric state (PDB 1GKO [36]; orange). Note that the last β-strand H in the X-ray model is disoriented in the NMR model [35]. (**b**) The NMR structural model of T119M M-TTR (the lowest energy conformer; blue) is shown with the X-ray model of M-TTR (orange). The last β-strand H, shown in the X-ray model, which is missing in the NMR model of M-TTR (a), is visible here, with a non-native β-strand structure [34]. (**c**) Comparison between the X-ray model of tetrameric M-TTR (orange) and the NMR model of monomeric T119M M-TTR (blue). Note that the sidechain of T119 in M-TTR is oriented outward, whereas the sidechain of M119 in T119M M-TTR is directed inward [34].

Structural features of TTR monomers were recently revealed using solution-state nuclear magnetic resonance (NMR) spectroscopy. Compared with X-ray crystallography, NMR spectroscopy is advantageous for investigating dynamic molecules in a solution state. By employing this technique, solution structural models of two monomeric variants, viz., TTR(F87M/L110M; M-TTR) and TTR(F87M/L110M/T119M; T119M M-TTR), were determined at an atomic resolution [34,35]. M-TTR was designed to stabilize the monomeric state of TTR [36], whereas the T119M substitution is a structure-stabilizing mutation that effectively suppresses the amyloidogenic property of TTR [37,38]. A previous X-ray crystallographic study of M-TTR showed that it tetramerized under crystallization conditions, and its tertiary structure was highly similar to that of wild-type (WT) TTR [36]. In contrast, NMR spectroscopy revealed that both M-TTR [35] and T119M M-TTR [34] stabilized to monomeric states with different secondary and tertiary structures (Figure 3). Notably, considering the highly dynamic characteristics of M-TTR [39], high-pressure NMR was used to reduce structural heterogeneity and improve the spectral quality [40]. Pressurized conditions were previously employed to investigate the intermediate tetrameric and monomeric states of TTR [41,42,43]. The NMR structural model of M-TTR was characterized by the loss of the final β-strand H, which forms a stable dimeric interface in the tetrameric state, and significant structural alteration of the FG loop (the loop between the β-strands F and G) (Figure 3a). In contrast, the NMR structural model of T119M M-TTR showed that the β-strand H was restored to the non-native conformation (Figure 3b) [34]. Compared to the X-ray structure of M-TTR in its tetrameric state, the β-strands G and H were shifted mainly due to reorientation of the sidechain of residue 119; the sidechain of T119 in M-TTR tetramer was oriented outward, whereas the sidechain of M119 in T119M M-TTR was directed inward, toward the hydrophobic interior, resulting in overall structural rearrangement (Figure 3c).

Notable implications of these results come from one of the initial studies that attempted to identify the amyloidogenic region of TTR. Gustavsson et al. found that the peptide originating from residues 105–115 of TTR, corresponding mostly to the β-strand G, was highly amyloidogenic [44]. Since then, amyloid fibrils made from this peptide have been used as a model to investigate their amyloidogenic features [45,46]. Therefore, this indicates that the β-strand H may play a protective role in minimizing exposure of the amyloidogenic β-strand G, whereas monomeric TTR cannot benefit from this protective effect, thus, becoming more prone to aggregation.

Another important implication of the solution structural studies of monomeric TTR is that monomeric TTR has fairly dynamic structural features, represented by the two different structural states of M-TTR and T119M M-TTR, and by the requirement of non-native pressurized conditions for determining the structure of M-TTR. Findings of investigations using various biochemical and biophysical approaches are consistent. For example, relaxation dispersion NMR experiments on M-TTR [39] and WT TTR [47] indicated that the DAGH β-sheet possessed considerable flexibility, and particular dynamic features were observed at the β-strand H. Solution NMR [48] and fluorescence spectroscopic [49] studies have proposed that the monomeric state of TTR may maintain the native-like CBEF β-sheet structure, whereas the structure of the DAGH β-sheet may experience significant perturbation in an aggregation-prone state. Recent molecular dynamics (MD) simulation results have consistently indicated that edge strands, such as the β-strands D and H, are susceptible to structural perturbation under amyloidogenic conditions [50]. Moreover, site-directed mutagenesis to identify the residues indispensable for amyloid formation provided evidence suggesting that β-strands F and H play an important role in TTR aggregation [51]. In the study, proline residues were artificially introduced in several regions of TTR, showing that the residues in β-strand F are essential for efficient amyloid formation. In addition, when the residues in β-strand H were substituted with bulky aromatic residues, such as T119W, T119Y, V121W, and V121Y, the propensity of TTR to aggregate was significantly reduced. Subsequently, a short peptide that was designed to bind to β-strands F and H was proven to act as a TTR aggregation inhibitor.

Intriguingly, it has also been suggested that the hydrogen-bond network and the sidechain packing around the β-strands F and H are critical for stabilizing the tetramer [52]. Yokoyama et al. used neutron crystallographic approaches to show that the network of hydrogen bonds consisting of T75, W79, H88, S112, P113, and T118, along with a few water molecules, is important for maintaining the tetrameric quaternary structure of TTR. They found that H88 was in a deprotonated state at pD 7.4, and was involved in the hydrogen-bond network, although the same residue was found to be in a protonated state based on the X-ray crystallographic model obtained at pH 4, the condition facilitating the aggregation of TTR. In their subsequent work, they found that the stability of TTR(H88R), the known variant associated with amyloid cardiomyopathy, is much less than that of WT; thus, it is more prone to aggregation [53]. They also confirmed that H88S substitution, which caused significant loss of the hydrogen-bond network around H88, resulted in tetramer destabilization and subsequent enhancement of the propensity to aggregate. Consistently, Sun et al., using ^19^F NMR spectroscopy, revealed that TTR with W79 fluorinated at the 6th position was less stable and more amyloidogenic than TTR with W79 fluorinated at the 5th position, indicating that the residue interaction around this region is indeed important for the quaternary stability of TTR [54].

Another intriguing point regarding the structural deformation of monomeric TTR is that α-sheets, transient secondary structures that have been observed in several amyloidogenic proteins [55,56], were proposed as intermediate structures of monomeric TTR in its aggregation pathway [57]. Steward et al. reported that amyloidogenic variants of TTR were able to manifest α-sheet structures in the aggregation-prone state [58]. More recently, MD simulation data have indicated that TTR tetramer dissociation facilitates the formation of α-sheet structures on the TTR monomer, which is followed by its aggregation [59]. Based on these observations, Daggett et al. designed α-sheet peptides that are complementary to α-sheet structures of amyloid-β and TTR, and confirmed their activity against amyloidosis [60,61], supporting the hypothesis that α-sheet structures are an intermediate state in the amyloidosis pathway.

### 2.2. TTR Dimers

A series of evidence indicates the presence of a dimeric state in the TTR tetramer dissociation pathway (Figure 2b). For example, Foss et al. reported that, among the two intermolecular interfaces, viz., the hydrophobic interface between the dimers AB and CD, and the extensively hydrogen-bonded interface between the dimers AC and BD (Figure 1a), the hydrophobic interface could be more vulnerable to denaturation, and the dimers AB and CD might be transiently present in the tetramer dissociation pathway [32]. Their results indicated that direct dissociation of monomeric species from the tetramer is unlikely. Consistently, TTR(S112I), the mutation causing amyloidosis with severe cardiomyopathy, has been reported to stabilize the dimeric state of TTR [62]. Structural investigation of this variant indicated that dimeric TTR(S112I) maintained WT-like secondary structures, yet failed to form WT-like T_4_-binding pockets. This observation is consistent with that of Foss et al. in that the dimeric species only maintain the hydrogen-bonded interface, not the hydrophobic interface [32]. Subsequent neutron crystallographic studies further confirmed that S112 of WT TTR is involved in forming a network of hydrogen bonds at the hydrophobic interface between AC and BD [52,63]. Notably, MD simulation study of S117E M-TTR (i.e., TTR[F87M/L110M/S117E]) showed that the tetrameric complex of this construct readily dissociated and formed the relatively stable AB and CD dimers [64]. Furthermore, a recent native mass-spectrometry and surface-induced dissociation study, following subunit exchange with untagged and tagged TTR proteins, revealed that the dimeric state is an intermediate for tetramers to be dissociated into monomers [65].

In contrast, a few reports also propose that the dimeric state can be formed as a subsequent intermediate after monomerization of TTR. For example, Olofsson et al. have shown that, after complete denaturation, TTR forms dimeric intermediates that are prone to further aggregation and amyloid formation [66]. More recently, Dasari et al. were able to obtain dimeric TTR by incubating WT TTR and TTR(G53A) at pH 4.4, at 4 °C, wherein TTR aggregation was slow enough to catch up with some intermediates [67]. In this study, TTR dimers were observed during the initial incubation period, and decreased gradually. Further, size-exclusion chromatography (SEC) indicated that TTR dimers were converted to hexameric oligomers, and subsequently to larger heterogeneous oligomers. By purifying the dimeric species, they acquired the circular dichroism spectra of the TTR dimer, indicating that the dimer becomes more disordered with decreased β-strand content than WT (34.5% in dimer vs. 42% in WT). Pires et al. employed atomic force microscopy (AFM) to investigate annular oligomers, which were frequently observed in the acid-induced aggregation mixture of TTR and found that these oligomers exhibited force spectroscopic features that were consistent with TTR dimers [68]. This observation suggests that at least some parts of the annular oligomers consist of dimeric subunits. Notably, the dimeric construct of TTR, viz., TTR(S112I), was also shown to form relatively small spherical oligomers at pH 7 and 37 °C [62], implying that TTR dimers may indeed act as building blocks for certain oligomers. Thus, it is evident that the dimeric species of TTR occurs as a transient state in tetramer dissociation or oligomerization pathways; however, further investigation is required to understand its structural and functional nature.

### 2.3. Oligomerization and Amyloid Formation

Small nonfibrillar oligomers have been observed with various TTR variants in the amyloid formation pathway, suggesting that these oligomers may be responsible for the significant cytotoxicity of TTR aggregates [69,70]. Investigation of TTR oligomers has been challenging due to their transient nature. Yet, there have been recent advancements in understanding their structural features. Faria et al. used SEC-coupled multiangle light scattering to reveal that transient TTR oligomers consisting of 6–10 monomers were formed in the acid-induced aggregation of TTR [71]. As discussed above, Dasari et al. also observed the formation of several oligomeric species in acid-induced aggregation experiments at 4 °C [67]. They used SEC analysis to purify the oligomeric species and analyzed them with circular dichroism to find that the secondary structural features of oligomers were more disordered than those of the native tetramers. Using solid-state NMR analysis, Dasari et al. further confirmed that, although certain regions remained rigid and unchanged, the oligomers exhibited a more extended disorder in their overall structure than the native tetramer. High-resolution AFM analysis by Pires et al. indicated that WT TTR formed annular oligomers of ~16 nm as early intermediates in the amyloidosis at pH 3.6 [72]. Intriguingly, subsequent detailed analysis of AFM images suggested that annular oligomers are likely composed of double stacks of octamers and exhibit a tendency to convert into spheroidal oligomers consisting of 8–16 monomers. In addition, Pires et al. observed that the protofibrils, linear fibrillar intermediates that were often formed from a soluble oligomer, showed a periodic structure, suggesting the presence of ~15-nm subunits. This observation indicated that the protofibril might be formed by the mechanism of adding 15-nm oligomers to the end of the protofibril [73]. More recently, Frangolho et al. used photo-induced crosslinking experiments to capture up to octameric species during acid-induced TTR aggregation at pH 3.6 [74]. They found that amyloidogenic variants of TTR showed faster oligomerization rates, and also provided evidence supporting sequential monomeric addition to oligomers.

In addition to structural studies on oligomers, significant advancements have been made in elucidating the structural details of TTR amyloid fibrils. Cryo-electron microscopy (cryo-EM) of TTR amyloid fibrils, collected from a patient suffering from hereditary TTR(V30M) amyloidosis, revealed that the structural features of TTR amyloid fibrils are fairly distinct from their native structure, suggesting severe structural alterations during TTR aggregation [75]. In comparison, solid-state NMR approaches have suggested that the native-like β-strand architectures of TTR are maintained at least in a part of amyloid fibrils that are formed under acidic conditions [76], along with local structural changes in the AB loop [48]. A subsequent study found that amyloid fibrils formed from amyloidogenic variants, such as V30M and L55P TTR, have distinctive structural states in the β-strands A and D [77]. This discrepancy between cryo-EM and solid-state NMR results, along with another solid-state NMR structural model of the amyloid fibrils made with TTR(105-115) peptides [78], needs to be further elucidated. Yet, it may indicate heterogeneous amyloidosis mechanisms of TTR that are likely to depend on factors such as genetic variations and conditions of aggregation.

## 3. Proteolysis-Induced Aggregation of TTR

In comparison to the above-mentioned mechanism of TTR amyloidosis, another mechanism involving proteolysis of TTR has recently attracted much attention due to its novel implications in the amyloidosis and pathogenesis of TTR (Figure 2c). The initial observation of truncated TTR in the amyloid fibril deposits dates back to the early 1990s [13,79,80], and subsequent analyses have revealed two distinct morphologies in TTR-originated amyloid fibrils, named as types A and B. It was shown that type B amyloid, having relatively long fibrils arranged in parallel bundles, is composed of full-length TTR, whereas the type A amyloid, having short, tightly packed, and unoriented fibrils, is composed of the C-terminal fragment of TTR [81]. Residues between 46 and 59, covering the β-strands C and D as well as the loop between them (the CD loop), were proposed as proteolytic sites [79,80,81,82].

Since then, many studies have been conducted to elucidate the related mechanisms, yet some conflicts regarding the mechanism of TTR proteolysis still persist. A recent cryo-EM study on patient-derived amyloid fibrils suggested that the initial fibrillization of TTR involves the full-length TTR, yet subsequent post-amyloidosis exposure of the region corresponding to the proteolytic sites results in the proteolysis of TTR and formation of truncated TTR species [75].

In comparison, a series of studies proposed that proteolysis plays a more direct role in TTR amyloidosis. For example, Mangione et al. showed that TTR(S52P), the variant that is responsible for aggressive and penetrant systemic amyloidosis, is susceptible to trypsin-mediated proteolysis causing the formation of the 49–127 fragment of TTR [33]. They conducted an X-ray crystallographic study with TTR(S52P) and observed that S52P substitution caused loss of the hydrogen-bond network between S50, S52, and E54, resulting in structural destabilization of the native tetrameric complex and increased susceptibility of the CD loop region to proteolytic attacks. Yee et al. also made consistent observations with MD simulation and neutron crystallography, suggesting that S52P substitution induced an altered hydrogen-bond network around the CD loop region [83]. A subsequent study by Marcoux et al. showed that enzymatic truncation and amyloid formation is more effective with mechanical perturbation, which might be analogous to the shear stress of TTR in the blood stream [84]. Marcoux et al. found that even after proteolytic cleavage, the cleaved N-terminal fragment remained bound to the native-like tetrameric complex. Therefore, it was suggested that application of biomechanical forces may be necessary for efficient dissociation of the N-terminal fragment and destabilization of the tetrameric state.

Multiple studies on additional TTR variants have provided consistent results. Klimtchuk et al. reported an unusual duplication mutation in which E51 and S52 were repeated twice in the primary structure of TTR [85]. They found that this variant became more susceptible to tryptic proteolysis due to the increased disorder in the CD loop region, resulting in the accumulation of TTR(49–127) fragments and subsequent aggregation. This observation was consistent with its aggressive pathogenic features and poor response to diflunisal treatment. More recently, Dasari et al. also made consistent observations with TTR(G53A), which produced TTR(49–127) by trypsin-mediated proteolysis [86]. They found that the solid-state NMR spectroscopic and FT-IR spectral features of the amyloid fibrils formed with full-length and truncated TTRs were similar and proposed that the cleavage at the CD loop destabilized the native tetrameric state, facilitating TTR monomer dissociation and aggregation.

Researchers have tried to identify a protease that is mainly responsible for TTR truncation, because trypsin may not be a probable candidate as it is secreted from the pancreas and may not likely encounter TTR in the plasma [84,87]. One protease candidate was plasmin, a serine protease that is activated from plasminogen and can dissolve fibrin blood clots. Mangione et al. found that WT TTR and amyloidogenic variants were highly susceptible to plasmin-mediated proteolysis, producing TTR(49–127) fragments, and confirmed that this cleavage efficiently caused significant TTR amyloidosis [88]. In contrast, it was also proposed that subtilisin, a serine protease from *Bacillus subtilis*, is responsible for TTR proteolysis and subsequent amyloidosis [87]. Subtilisin was shown to produce the amyloidogenic fragment TTR(59–127). *B. subtilis* is a nonpathogenic microbe in the gut, and increased permeability of the intestinal mucosa with aging was proposed as a working mechanism for supplying subtilisin to human plasma.

Notably, this novel aggregation mechanism raised the question whether the therapeutic strategy based on the kinetic stabilizer for tetramer stabilization to prevent TTR aggregation will be effective in inhibiting proteolysis and subsequent aggregation of TTR. A recent study by Marcoux et al. showed that kinetic stabilizers, such as tafamidis, diflunisal, and mds84, are effective in preventing proteolysis and subsequent fibrillization of TTR [84]. Intriguingly, the inhibitory activity of these molecules against TTR proteolysis depended on their valencies; monovalent ligands (tafamidis and diflunisal) were less effective than mds84, a palindromic divalent ligand occupying both T_4_-binding sites of TTR simultaneously [89]. This result suggests that even though one molecule of a monovalent ligand binds to one T_4_-binding pocket of the TTR tetramer, monomeric subunits of the other unoccupied binding site may not be protected against proteolysis. The ineffectiveness of diflunisal for amyloidosis treatment trial against the E51-S52 duplication variant of TTR may support this hypothesis [85]. In addition, Corazza et al. performed a solution NMR spectroscopic study, and observed that monovalent ligands caused limited NMR signal perturbation, whereas a more extensive signal perturbation pattern was achieved with a divalent ligand [90]. Several ligand molecules, such as T_4_, resveratrol, and tafamidis, were reported to bind to the two T_4_-binding sites with negative cooperativity; upon occupation of the first binding site, the second binding site exhibited significantly reduced affinity for a ligand molecule [26,31,90,91,92]. Therefore, in most cases, it is expected that only one binding pocket of TTR is occupied, suggesting that TTR subunits that are not directly benefited by ligand-induced stabilization may still be vulnerable to proteolysis.

Intriguingly, studies testing the susceptibility of TTR variants to proteolysis indicated that the T119M variant, which increases the overall stability of TTR structures, and thus, inhibits TTR aggregation, is less vulnerable to proteases than WT TTR [84,88]. This indicates that the T119M substitution may induce structural changes in the proteolysis-sensitive regions, i.e., the residues around 46–59. A solution NMR spectroscopic study showed that T119M substitution induced significant signal shifts around the CD loop region [93]. In addition, studies on ligand-bound TTR complexes yielded consistent findings; tafamidis and mds84 shifted the TTR signals corresponding to the residues 50–55 significantly, and the intensity of perturbation was higher in mds84 [90,94]. Along with the resistance against proteolysis acquired by ligand binding, these observations imply that some tetramer stabilization mechanisms of TTR are coupled with structural changes, making the proteolysis-sensitive region of TTR less vulnerable.

## 4. Conclusions

Since its discovery in the 1950s, numerous structural studies have been conducted to evaluate the functional and pathological features of TTR. Initial X-ray crystallographic studies focused on revealing the atomic-resolution features of the TTR tetramer. These studies provide critical information for understanding the physiological features of TTR in its native state, and for designing therapeutic molecules, such as tafamidis and diflunisal, that can bind to the T_4_-binding pocket and stabilize the tetrameric complex, thus, reducing amyloidogenic propensity [26,30]. In recent years, advancements have been made in understanding the structural features of non-native and misfolded monomeric, oligomeric, and aggregated TTR species, employing various interdisciplinary techniques, such as solution/solid-state NMR spectroscopy, force spectroscopy, mass spectrometry, cryo-EM, and computational modeling. In particular, the proteolysis-induced amyloidosis mechanism of TTR has attracted much attention in recent years, and various structural studies have revealed the mechanistic details of this amyloidosis process [95]. Along with recent boosts in multidisciplinary methodology developments, further improvements are soon expected in understanding of TTR amyloidosis pathology and developing an even more effective therapeutic strategy against this disease.

Despite these remarkable advances, a few ambiguities still persist, and require further investigation. First, the mechanistic details of TTR amyloidosis remain to be further elucidated. It is evident that the chemical/physical instability of TTR tetramers is related to amyloidosis, yet accumulated evidence strongly suggests that there are multiple pathways for structural deformation and subsequent amyloidosis, depending on various factors, such as genetic mutations, cellular conditions, and proteolytic stress. Consistently, it appears that several conflicting observations regarding morphological features of TTR aggregates could be explained by heterogenous aggregation mechanisms of TTR. Indeed, it was previously reported that different TTR variants might experience differential structural deformation pathways that could be responsible for amyloidosis or cytotoxic activities [96,97]. Moreover, ex vivo cardiac fibrils from a human patient could work as a seed to facilitate TTR fibrillization even at physiological pH, raising the intriguing possibility that TTR amyloidosis may also involve nucleation, as many other amyloidogenic proteins do [98]. In contrast, small-angle X-ray scattering experiments by Groenning et al. indicated that protofibril-like species could be exclusively formed from monomeric subunits [99]. As this observation was made at pH ~3, it is probable that TTR amyloidosis may be facilitated by a distinctive mechanism under different conditions. Notably, there were several consistent reports that some natural compounds, such as epigallocatechin gallate and the related flavonoids, could inhibit TTR amyloidosis without occupying the T_4_ hydrophobic binding pockets [100,101,102], suggesting that the working mechanism of these small molecules may differ from that of kinetic stabilizers. This raises an intriguing possibility that TTR may have multiple aggregation pathways exhibiting differential susceptibilities to different compounds.

Structural elucidation of TTR at various intermediate states is necessary to understand the structural mechanism of TTR amyloidosis in detail. For example, although the monomeric structure of M-TTR was revealed using NMR spectroscopy, several reports indicate that the actual structure of WT TTR in its monomeric amyloidogenic state is different from that of M-TTR [36]. SEC of aged TTR samples indicated that monomeric TTR could be converted into still monomeric, yet structurally different species during TTR amyloidosis [103]. In addition, elucidating the detailed structural features of TTR dimers and oligomers is important, as it may provide critical insights into the aggregation mechanism of TTR and help in identifying novel targets that can be used as points for therapeutic intervention. It is also critical to investigate the heterogeneous structural features of ligand-bound TTR, because it can give detailed pictures revealing the antiaggregation mechanism of therapeutic ligands. Notable example includes the negative cooperativity of a few ligand molecules for the two T_4_ binding pockets; atomistic details of this mechanism may contribute much to understand how ligands modulate the tetrameric stability of TTR and exert their therapeutic effects.

Revealing the mechanistic details of the proteolysis-mediated misfolding of TTR is crucial. It is evident that proteolysis induces quaternary instability of the TTR structure. Yet, it is still very challenging to identify the structural perturbation caused by proteolysis, and how it affects the formation of subsequent dimeric or oligomeric intermediates. It is required to identify which proteases are responsible for TTR fragmentation, which may need to consider a tissue-specific protease profile, as proteolytic activity and a responsible protease may differ between, e.g., plasma and CSF. In addition, consideration regarding heterogeneous fragmentation of TTR, such as relative populations of TTR(49-127) vs. TTR(59-127) in a physiological condition, and probable involvement of full-length TTR need to be made.

## References

[1] Ingbar S.H. (1958). Pre-albumin: A thyroxine-binding protein of human plasma. Endocrinology.

[2] Robbins J., Rall J.E. (1960). Proteins associated with the thyroid hormones. Physiol. Rev..

[3] Ingbar S.H. (1963). Observations concerning the binding of thyroid hormones by human serum prealbumin. J. Clin. Investig..

[4] Stabilini R., Vergani C., Agostoni A., Agostoni R.P.V. (1968). Influence of age and sex on prealbumin levels. Clin. Chim. Acta.

[5] Schreiber G., Aldred A.R., Jaworowski A., Nilsson C., Achen M.G., Segal M.B. (1990). Thyroxine transport from blood to brain via transthyretin synthesis in choroid plexus. Am. J. Physiol. Integr. Comp. Physiol..

[6] Palha J.A. (2002). Transthyretin as a thyroid hormone carrier: Function revisited. Clin. Chem. Lab. Med..

[7] Kato M., Kato K., Blaner W.S., Chertow B.S., Goodman D.S. (1985). Plasma and cellular retinoid-binding proteins and transthyretin (prealbumin) are all localized in the islets of Langerhans in the rat. Proc. Natl. Acad. Sci. USA.

[8] Pfeffer B.A., Becerra S.P., Borst D.E., Won P. (2004). Expression of transthyretin and retinol binding protein mRNAs and secretion of transthyretin by cultured monkey retinal pigment epithelium. Mol. Vis..

[9] Blake C.C.F., Swan I.D.A., Rerat C., Berthou J., Laurent A., Rerat B. (1971). An X-ray study of the subunit structure of prealbumin. J. Mol. Biol..

[10] Blake C.C.F., Geisow M.J., Oatley S.J., Rérat B., Rérat C. (1978). Structure of prealbumin: Secondary, tertiary and quaternary interactions determined by Fourier refinement at 1.8 Å. J. Mol. Biol..

[11] Wojtczak A., Cody V., Luft J.R., Pangborn W. (1996). Structures of human transthyretin complexed with thyroxine at 2.0 Å resolution and 3′,5′-dinitro-N-acetyl-L-thyronine at 2.2 Å resolution. Acta Crystallogr. Sect. D Biol. Crystallogr..

[12] Costa P.P., Figueira A.S., Bravo F.R. (1978). Amyloid fibril protein related to prealbumin in familial amyloidotic polyneuropathy. Proc. Natl. Acad. Sci. USA.

[13] Westermark P., Sletten K., Johansson B., Cornwell G.G. (1990). Fibril in senile systemic amyloidosis is derived from normal transthyretin. Proc. Natl. Acad. Sci. USA.

[14] Coelho T. (1996). Familial amyloid polyneuropathy: New developments in genetics and treatment. Curr. Opin. Neurol..

[15] Jacobson D.R., Pastore R.D., Yaghoubian R., Kane I., Gallo G., Buck F.S., Buxbaum J.N. (1997). Variant-sequence transthyretin (isoleucine 122) in late-onset cardiac amyloidosis in black Americans. N. Engl. J. Med..

[16] Connors L.H., Lim A., Prokaeva T., Roskens V.A., Costello C.E. (2003). Tabulation of human transthyretin (TTR) variants, 2003. Amyloid.

[17] Jacobson D.R., Alexander A.A., Tagoe C., Buxbaum J.N. (2015). Prevalence of the amyloidogenic transthyretin (TTR) V122I allele in 14 333 African–Americans. Amyloid.

[18] Buxbaum J.N., Ruberg F.L. (2017). Transthyretin V122I (pV142I)* cardiac amyloidosis: An age-dependent autosomal dominant cardiomyopathy too common to be overlooked as a cause of significant heart disease in elderly African Americans. Genet. Med..

[19] Akinboboye O., Shah K., Warner A.L., Damy T., Taylor H.A., Gollob J., Powell C., Karsten V., Vest J., Maurer M.S. (2020). DISCOVERY: Prevalence of transthyretin (*TTR*) mutations in a US-centric patient population suspected of having cardiac amyloidosis. Amyloid.

[20] Johnson S.M., Connelly S., Fearns C., Powers E.T., Kelly J.W. (2012). The transthyretin amyloidoses: From delineating the molecular mechanism of aggregation linked to pathology to a regulatory-agency-approved drug. J. Mol. Biol..

[21] Colon W., Kelly J.W. (1992). Partial denaturation of transthyretin is sufficient for amyloid fibril formation in vitro. Biochemistry.

[22] Lai Z., Colón W., Kelly J.W. (1996). The acid-mediated denaturation pathway of transthyretin yields a conformational intermediate that can self-assemble into amyloid. Biochemistry.

[23] Miroy G.J., Lai Z., Lashuel H.A., Peterson S.A., Strang C., Kelly J.W. (1996). Inhibiting transthyretin amyloid fibril formation via protein stabilization. Proc. Natl. Acad. Sci. USA.

[24] Razavi H., Palaninathan S.K., Powers E.T., Wiseman R.L., Purkey H.E., Mohamedmohaideen N.N., Deechongkit S., Chiang K.P., Dendle M.T.A., Sacchettini J.C. (2003). Benzoxazoles as transthyretin amyloid fibril inhibitors: Synthesis, evaluation, and mechanism of action. Angew. Chemie Int. Ed..

[25] Johnson S.M., Wiseman R.L., Sekijima Y., Green N.S., Adamski-Werner S.L., Kelly J.W. (2005). Native state kinetic stabilization as a strategy to ameliorate protein misfolding diseases: A focus on the transthyretin amyloidoses. Acc. Chem. Res..

[26] Bulawa C.E., Connelly S., DeVit M., Wang L., Weigel C., Fleming J.A., Packman J., Powers E.T., Wiseman R.L., Foss T.R. (2012). Tafamidis, a potent and selective transthyretin kinetic stabilizer that inhibits the amyloid cascade. Proc. Natl. Acad. Sci. USA.

[27] Le Bras A. (2018). Tafamidis: A new treatment for ATTR cardiomyopathy. Nat. Rev. Cardiol..

[28] Burton A., Castaño A., Bruno M., Riley S., Schumacher J., Sultan M.B., See Tai S., Judge D.P., Patel J.K., Kelly J.W. (2021). Drug Discovery and Development in Rare Diseases: Taking a Closer Look at the Tafamidis Story. Drug Des. Devel. Ther..

[29] Hörnberg A., Eneqvist T., Olofsson A., Lundgren E., Sauer-Eriksson A.E. (2000). A comparative analysis of 23 structures of the amyloidogenic protein transthyretin. J. Mol. Biol..

[30] Palaninathan S.K. (2012). Nearly 200 X-ray crystal structures of transthyretin: What do they tell us about this protein and the design of drugs for TTR amyloidoses?. Curr. Med. Chem..

[31] Zanotti G., Vallese F., Ferrari A., Menozzi I., Saldaño T.E., Berto P., Fernandez-Alberti S., Berni R. (2017). Structural and dynamics evidence for scaffold asymmetric flexibility of the human transthyretin tetramer. PLoS ONE.

[32] Foss T.R., Wiseman R.L., Kelly J.W. (2005). The pathway by which the tetrameric protein transthyretin dissociates. Biochemistry.

[33] Mangione P.P., Porcari R., Gillmore J.D., Pucci P., Monti M., Porcari M., Giorgetti S., Marchese L., Raimondi S., Serpell L.C. (2014). Proteolytic cleavage of Ser52Pro variant transthyretin triggers its amyloid fibrillogenesis. Proc. Natl. Acad. Sci. USA.

[34] Kim J.H., Oroz J., Zweckstetter M. (2016). Structure of monomeric transthyretin carrying the clinically important T119M mutation. Angew. Chemie Int. Ed..

[35] Oroz J., Kim J.H., Chang B.J., Zweckstetter M. (2017). Mechanistic basis for the recognition of a misfolded protein by the molecular chaperone Hsp90. Nat. Struct. Mol. Biol..

[36] Jiang X., Smith C.S., Petrassi H.M., Hammarström P., White J.T., Sacchettini J.C., Kelly J.W. (2001). An engineered transthyretin monomer that is nonamyloidogenic, unless it is partially denatured. Biochemistry.

[37] Coelho T., Carvalho M., Saraiva M., Alves C., Almeida M., Costa P. (1993). A strikingly benign evolution of FAP in an individual found to be a compound heterozygote for two TTR mutations: TTR Met30 and TTR Met119. J. Rheumatol..

[38] Hammarström P., Schneider F., Kelly J.W. (2001). Trans-suppression of misfolding in an amyloid disease. Science.

[39] Lim K.H., Dyson H.J., Kelly J.W., Wright P.E. (2013). Localized structural fluctuations promote amyloidogenic conformations in transthyretin. J. Mol. Biol..

[40] Roche J., Royer C.A., Roumestand C. (2017). Monitoring protein folding through high pressure NMR spectroscopy. Prog. Nucl. Magn. Reson. Spectrosc..

[41] Ferrão-Gonzales A.O., Souto S.O., Silva J.L., Foguel D. (2000). The preaggregated state of an amyloidogenic protein: Hydrostatic pressure converts native transthyretin into the amyloidogenic state. Proc. Natl. Acad. Sci. USA.

[42] Foguel D., Suarez M.C., Ferrão-Gonzales A.D., Porto T.C.R., Palmieri L., Einsiedler C.M., Andrade L.R., Lashuel H.A., Lansbury P.T., Kelly J.W. (2003). Dissociation of amyloid fibrils of α-synuclein and transthyretin by pressure reveals their reversible nature and the formation of water-excluded cavities. Proc. Natl. Acad. Sci. USA.

[43] Palhano F.L., Leme L.P., Busnardo R.G., Foguel D. (2009). Trapping the monomer of a non-amyloidogenic variant of transthyretin: Exploring its possible use as a therapeutic strategy against transthyretin amyloidogenic diseases. J. Biol. Chem..

[44] Gustavsson Å., Engström U., Westermark P. (1991). Normal transthyretin and synthetic transthyretin fragments from amyloid-like fibrils in vitro. Biochem. Biophys. Res. Commun..

[45] Liang Y., Ore M.O., Morin S., Wilson D.J. (2012). Specific disruption of transthyretin(105-115) fibrilization using “stabilizing” inhibitors of transthyretin amyloidogenesis. Biochemistry.

[46] Fitzpatrick A.W.P., Debelouchina G.T., Bayro M.J., Clare D.K., Caporini M.A., Bajaj V.S., Jaroniec C.P., Wang L., Ladizhansky V., Müller S.A. (2013). Atomic structure and hierarchical assembly of a cross-β amyloid fibril. Proc. Natl. Acad. Sci. USA.

[47] Das J.K., Mall S.S., Bej A., Mukherjee S. (2014). Conformational flexibility tunes the propensity of transthyretin to form fibrils through non-native intermediate states. Angew. Chemie Int. Ed..

[48] Lim K.H., Dasari A.K.R., Hung I., Gan Z., Kelly J.W., Wemmer D.E. (2016). Structural changes associated with transthyretin misfolding and amyloid formation revealed by solution and solid-state NMR. Biochemistry.

[49] Jazaj D., Ghadami S.A., Bemporad F., Chiti F. (2019). Probing conformational changes of monomeric transthyretin with second derivative fluorescence. Sci. Rep..

[50] Childers M.C., Daggett V. (2020). Edge strand dissociation and conformational changes in transthyretin under amyloidogenic conditions. Biophys. J..

[51] Saelices L., Johnson L.M., Liang W.Y., Sawaya M.R., Cascio D., Ruchala P., Whitelegge J., Jiang L., Riek R., Eisenberg D.S. (2015). Uncovering the mechanism of aggregation of human transthyretin. J. Biol. Chem..

[52] Yokoyama T., Mizuguchi M., Nabeshima Y., Kusaka K., Yamada T., Hosoya T., Ohhara T., Kurihara K., Tomoyori K., Tanaka I. (2012). Hydrogen-bond network and pH sensitivity in transthyretin: Neutron crystal structure of human transthyretin. J. Struct. Biol..

[53] Yokoyama T., Hanawa Y., Obita T., Mizuguchi M. (2017). Stability and crystal structures of His88 mutant human transthyretins. FEBS Lett..

[54] Sun X., Dyson H.J., Wright P.E. (2017). Fluorotryptophan incorporation modulates the structure and stability of transthyretin in a site-specific manner. Biochemistry.

[55] Daggett V. (2006). α-sheet: The toxic conformer in amyloid diseases?. Acc. Chem. Res..

[56] Bi T.M., Daggett V. (2018). The role of α-sheet in amyloid oligomer aggregation and toxicity. Yale J. Biol. Med..

[57] Armen R.S., Alonso D.O.V., Daggett V. (2004). Anatomy of an amyloidogenic intermediate: Conversion of β-sheet to α-sheet structure in transthyretin at acidic pH. Structure.

[58] Steward R.E., Armen R.S., Daggett V. (2008). Different disease-causing mutations in transthyretin trigger the same conformational conversion. Protein Eng. Des. Sel..

[59] Childers M.C., Daggett V. (2019). Drivers of α-sheet formation in transthyretin under amyloidogenic conditions. Biochemistry.

[60] Hopping G., Kellock J., Barnwal R.P., Law P., Bryers J., Varani G., Caughey B., Daggett V. (2014). Designed α-sheet peptides inhibit amyloid formation by targeting toxic oligomers. Elife.

[61] Maris N.L., Shea D., Bleem A., Bryers J.D., Daggett V. (2018). Chemical and physical variability in structural isomers of an L/D α-sheet peptide designed to inhibit amyloidogenesis. Biochemistry.

[62] Matsubara K., Mizuguchi M., Igarashi K., Shinohara Y., Takeuchi M., Matsuura A., Saitoh T., Mori Y., Shinoda H., Kawano K. (2005). Dimeric transthyretin variant assembles into spherical neurotoxins. Biochemistry.

[63] Mizuguchi M., Yokoyama T., Nabeshima Y., Kawano K., Tanaka I., Niimura N. (2012). Quaternary structure, aggregation and cytotoxicity of transthyretin. Amyloid.

[64] Dongmo Foumthuim C.J., Corazza A., Berni R., Esposito G., Fogolari F. (2018). Dynamics and thermodynamics of transthyretin association from molecular dynamics simulations. Biomed. Res. Int..

[65] Shirzadeh M., Boone C.D., Laganowsky A., Russell D.H. (2019). Topological analysis of transthyretin disassembly mechanism: Surface-induced dissociation reveals hidden reaction pathways. Anal. Chem..

[66] Olofsson A., Ippel H.J., Baranov V., Hörstedt P., Wijmenga S., Lundgren E. (2001). Capture of a dimeric intermediate during transthyretin amyloid formation. J. Biol. Chem..

[67] Dasari A.K.R., Hughes R.M., Wi S., Hung I., Gan Z., Kelly J.W., Lim K.H. (2019). Transthyretin aggregation pathway toward the formation of distinct cytotoxic oligomers. Sci. Rep..

[68] Pires R.H., Saraiva M.J., Damas A.M., Kellermayer M.S.Z. (2017). Force spectroscopy reveals the presence of structurally modified dimers in transthyretin amyloid annular oligomers. J. Mol. Recognit..

[69] Mendes Sousa M.M., Cardoso I., Fernandes R., Guimarães A., João Saraiva M. (2001). Deposition of transthyretin in early stages of familial amyloidotic polyneuropathy: Evidence for toxicity of nonfibrillar aggregates. Am. J. Pathol..

[70] Reixach N., Deechongkit S., Jiang X., Kelly J.W., Buxbaum J.N. (2004). Tissue damage in the amyloidoses: Transthyretin monomers and nonnative oligomers are the major cytotoxic species in tissue culture. Proc. Natl. Acad. Sci. USA.

[71] Faria T.Q., Almeida Z.L., Cruz P.F., Jesus C.S.H., Castanheira P., Brito R.M.M. (2015). A look into amyloid formation by transthyretin: Aggregation pathway and a novel kinetic model. Phys. Chem. Chem. Phys..

[72] Pires R.H., Karsai Á., Saraiva M.J., Damas A.M., Kellermayer M.S.Z. (2012). Distinct annular oligomers captured along the assembly and disassembly pathways of transthyretin amyloid protofibrils. PLoS ONE.

[73] Pires R.H., Saraiva M.J., Damas A.M., Kellermayer M.S.Z. (2011). Structure and assembly-disassembly properties of wild-type transthyretin amyloid protofibrils observed with atomic force microscopy. J. Mol. Recognit..

[74] Frangolho A., Correia B.E., Vaz D.C., Almeida Z.L., Brito R.M.M. (2020). Oligomerization profile of human transthyretin variants with distinct amyloidogenicity. Molecules.

[75] Schmidt M., Wiese S., Adak V., Engler J., Agarwal S., Fritz G., Westermark P., Zacharias M., Fändrich M. (2019). Cryo-EM structure of a transthyretin-derived amyloid fibril from a patient with hereditary ATTR amyloidosis. Nat. Commun..

[76] Lim K.H., Dasari A.K.R., Hung I., Gan Z., Kelly J.W., Wright P.E., Wemmer D.E. (2016). Solid-state NMR studies reveal native-like β-sheet structures in transthyretin amyloid. Biochemistry.

[77] Lim K.H., Dasari A.K.R., Ma R., Hung I., Gan Z., Kelly J.W., Fitzgerald M.C. (2017). Pathogenic mutations induce partial structural changes in the native β-sheet structure of transthyretin and accelerate aggregation. Biochemistry.

[78] Jaroniec C.P., MacPhee C.E., Bajaj V.S., McMahon M.T., Dobson C.M., Griffin R.G. (2004). High-resolution molecular structure of a peptide in an amyloid fibril determined by magic angle spinning NMR spectroscopy. Proc. Natl. Acad. Sci. USA.

[79] Thylen C., Wahlqvist J., Haettner E., Sandgren O., Holmgren G., Lundgren E. (1993). Modifications of transthyretin in amyloid fibrils: Analysis of amyloid from homozygous and heterozygous individuals with the Met30 mutation. EMBO J..

[80] Hermansen L.F., Bergman T., Jörnvall H., Husby G., Ranløv I., Sletten K. (1995). Purification and characterization of amyloid-related transthyretin associated with familial amyloidotic cardiomyopathy. Eur. J. Biochem..

[81] Bergström J., Gustavsson Å., Hellman U., Sletten K., Murphy C.L., Weiss D.T., Solomon A., Olofsson B.O., Westermark P. (2005). Amyloid deposits in transthyretin-derived amyloidosis: Cleaved transthyretin is associated with distinct amyloid morphology. J. Pathol..

[82] Kingsbury J.S., Théberge R., Karbassi J.A., Lim A., Costello C.E., Connors L.H. (2007). Detailed structural analysis of amyloidogenic wild-type transthyretin using a novel purification strategy and mass spectrometry. Anal. Chem..

[83] Yee A.W., Aldeghi M., Blakeley M.P., Ostermann A., Mas P.J., Moulin M., de Sanctis D., Bowler M.W., Mueller-Dieckmann C., Mitchell E.P. (2019). A molecular mechanism for transthyretin amyloidogenesis. Nat. Commun..

[84] Marcoux J., Mangione P.P., Porcari R., Degiacomi M.T., Verona G., Taylor G.W., Giorgetti S., Raimondi S., Sanglier-Cianférani S., Benesch J.L. (2015). A novel mechano-enzymatic cleavage mechanism underlies transthyretin amyloidogenesis. EMBO Mol. Med..

[85] Klimtchuk E.S., Prokaeva T., Frame N.M., Abdullahi H.A., Spencer B., Dasari S., Cui H., Berk J.L., Kurtin P.J., Connors L.H. (2018). Unusual duplication mutation in a surface loop of human transthyretin leads to an aggressive drug-resistant amyloid disease. Proc. Natl. Acad. Sci. USA.

[86] Dasari A.K.R., Arreola J., Michael B., Griffin R.G., Kelly J.W., Lim K.H. (2020). Disruption of the CD loop by enzymatic cleavage promotes the formation of toxic transthyretin oligomers through a common transthyretin misfolding pathway. Biochemistry.

[87] Peterle D., Pontarollo G., Spada S., Brun P., Palazzi L., Sokolov A.V., Spolaore B., Polverino de Laureto P., Vasilyev V.B., Castagliuolo I. (2020). A serine protease secreted from *Bacillus subtilis* cleaves human plasma transthyretin to generate an amyloidogenic fragment. Commun. Biol..

[88] Mangione P.P., Verona G., Corazza A., Marcoux J., Canetti D., Giorgetti S., Raimondi S., Stoppini M., Esposito M., Relini A. (2018). Plasminogen activation triggers transthyretin amyloidogenesis in vitro. J. Biol. Chem..

[89] Kolstoe S.E., Mangione P.P., Bellotti V., Taylor G.W., Tennent G.A., Deroo S., Morrison A.J., Cobb A.J.A., Coyne A., McCammon M.G. (2010). Trapping of palindromic ligands within native transthyretin prevents amyloid formation. Proc. Natl. Acad. Sci. USA.

[90] Corazza A., Verona G., Waudby C.A., Mangione P.P., Bingham R.P., Uings I., Canetti D., Nocerino P., Taylor G.W., Pepys M.B. (2019). Binding of monovalent and bivalent ligands by transthyretin causes different short- and long-distance conformational changes. J. Med. Chem..

[91] Ferguson R.N., Edelhoch H., Saroff H.A., Robbins J., Cahnmann H.J. (1975). Negative cooperativity in the binding of thyroxine to human serum prealbumin. Biochemistry.

[92] Florio P., Folli C., Cianci M., Del Rio D., Zanotti G., Berni R. (2015). Transthyretin binding heterogeneity and antiamyloidogenic activity of natural polyphenols and their metabolites. J. Biol. Chem..

[93] Liu K., Kelly J.W., Wemmer D.E. (2002). Native state hydrogen exchange study of suppressor and pathogenic variants of transthyretin. J. Mol. Biol..

[94] Liu Y.T., Yen Y.J., Ricardo F., Chang Y., Wu P.H., Huang S.J., Lin K.P., Yu T.Y. (2019). Biophysical characterization and modulation of Transthyretin Ala97Ser. Ann. Clin. Transl. Neurol..

[95] Bezerra F., Saraiva M.J., Almeida M.R. (2020). Modulation of the mechanisms driving transthyretin amyloidosis. Front. Mol. Neurosci..

[96] Leach B.I., Zhang X., Kelly J.W., Dyson H.J., Wright P.E. (2018). NMR measurements reveal the structural basis of transthyretin destabilization by pathogenic mutations. Biochemistry.

[97] Sun X., Jane Dyson H., Wright P.E. (2018). Kinetic analysis of the multistep aggregation pathway of human transthyretin. Proc. Natl. Acad. Sci. USA.

[98] Saelices L., Chung K., Lee J.H., Cohn W., Whitelegge J.P., Benson M.D., Eisenberg D.S. (2018). Amyloid seeding of transthyretin by ex vivo cardiac fibrils and its inhibition. Proc. Natl. Acad. Sci. USA.

[99] Groenning M., Campos R.I., Hirschberg D., Hammarström P., Vestergaard B. (2015). Considerably unfolded transthyretin monomers preceed and exchange with dynamically structured amyloid protofibrils. Sci. Rep..

[100] Miyata M., Sato T., Kugimiya M., Sho M., Nakamura T., Ikemizu S., Chirifu M., Mizuguchi M., Nabeshima Y., Suwa Y. (2010). The Crystal Structure of the Green Tea Polyphenol (−)-Epigallocatechin Gallate−Transthyretin Complex Reveals a Novel Binding Site Distinct from the Thyroxine Binding Site. Biochemistry.

[101] Ferreira N., Pereira-Henriques A., Almeida M.R. (2015). Transthyretin chemical chaperoning by flavonoids: Structure-activity insights towards the design of potent amyloidosis inhibitors. Biochem. Biophys. Rep..

[102] Ortore G., Orlandini E., Braca A., Ciccone L., Rossello A., Martinelli A., Nencetti S. (2016). Targeting Different Transthyretin Binding Sites with Unusual Natural Compounds. ChemMedChem.

[103] Quintas A., Vaz D.C., Cardoso I., Saraiva M.J.M., Brito R.M.M. (2001). Tetramer dissociation and monomer partial unfolding precedes protofibril formation in amyloidogenic transthyretin variants. J. Biol. Chem..

